# Innovative Approaches to Radiation Treatment for Mycosis Fungoides in the Setting of Collagen Vascular Disease

**DOI:** 10.1155/2015/853823

**Published:** 2015-08-27

**Authors:** Stephanie A. Terezakis, George C. Bohle, Ying-Chun Lo, Sean L. Berry, Joachim Yahalom

**Affiliations:** ^1^Department of Radiation Oncology and Molecular Radiation Sciences, Johns Hopkins Hospital, Baltimore, MD 21287, USA; ^2^Department of Radiation Oncology, Memorial Sloan-Kettering Cancer Center, New York, NY 10065, USA; ^3^Dental Service, Department of Surgery, Memorial Sloan-Kettering Cancer Center, New York, NY 10065, USA; ^4^College of Dentistry, University of Oklahoma, Oklahoma City, OK 73117, USA; ^5^Department of Oncology, Johns Hopkins School of Medicine, Baltimore, MD 21287, USA; ^6^Department of Pathology, Yale-New Haven Hospital, New Haven, CT 06510, USA

## Abstract

Patients with connective tissue disorders are clinically challenging for radiation oncologists as these patients may be at increased risk for radiation-related skin toxicity. A clinical dilemma presents itself in a patient with lupus who presents with confluent skin lesions from mycosis fungoides requiring radiotherapy. In this report, we discuss an innovative technique used to develop an immobilization device that also effectively functioned as a uniform bolus with distinct dosimetric advantages to the use of a facial moulage.

## 1. Introduction

Patients with connective tissue disorders present a formidable clinical challenge to the treating radiation oncologist as numerous reports have suggested that these patients may be at an increased risk of toxicity secondary to radiation treatment (RT) [1–6]. It has been hypothesized that patients with connective tissue disorder have increased radiosensitivity and possibly increased risk of pathological damage to the microvasculature from radiation therapy [7, 8]. Due to prior published case reports describing enhanced radiation-induced toxicity in these patients, it is likely that radiation is not offered to all those who may benefit even though the extent of these risks is still largely unknown [9]. Therefore, in current clinical practice, lupus is a relative but not an absolute contraindication of radiation therapy and radiation is only delivered when absolutely necessary [10]. Rather than avoiding radiation as a therapeutic modality, these patients may benefit from treatment techniques that minimize the risk of toxicity and maximize therapeutic benefit. We were presented with a difficult case of a patient with erythematous lupus and mycosis fungoides (MF) of the bilateral face. The patient had already progressed through known topical therapies and there were no additional treatment options that would address her facial skin lesions except radiation therapy. Current widely available techniques for treatment include combining the use of a normal thermoplastic mask for immobilization with supplemental bolus material (e.g., SuperFlab or PlayDoh). However, during the course of daily treatments that could extend over several weeks, it becomes challenging to guarantee the reproducibility of the bolus position, the daily adherence of the bolus to the patient's surface over the entire treatment area, and the integrity of the bolus material itself. The face contains many topographical variations and poorly fit bolus materials will degrade the delivered dose distribution, particularly at the patient's skin surface [11]. We describe and evaluate the clinical feasibility of a novel approach to creating an immobilization device that also functions as a bolus material. This device is customized and form-fitted using a digital model of the patient.

## 2. Case Report

**Figure 1:**
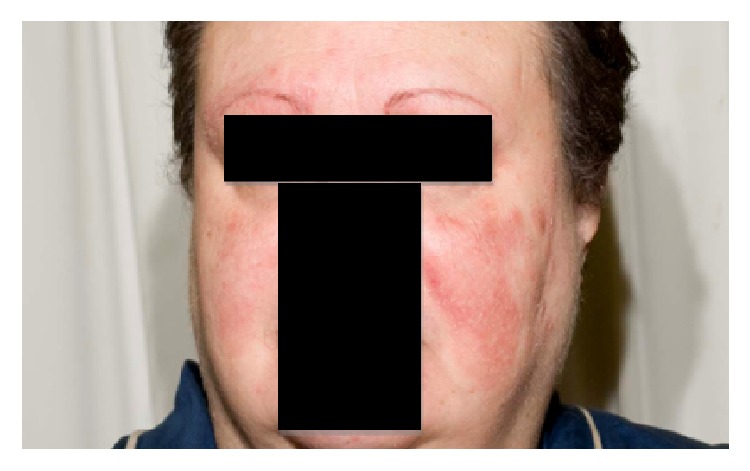
Pretreatment extent of clinically apparent disease.

This 60-year-old patient with history of erythematous lupus presented with a confluent facial eruption of classical (epidermotropic) MF and was referred to radiation oncology after several courses of topical therapies failed (Figure 1). Before being referred to radiation oncology, the patient received ultraviolet radiation therapy with no improvement. There were multiple lesions on the face beyond the very obvious lesion on the left cheek. There were multiple pathologic skin biopsies throughout the face performed which confirmed mycosis fungoides. Her treatment plan needed to target the disease sites on the surface of the face including the bilateral eyebrows, eyelids, cheeks, and temples. Given this patient's history of erythematous lupus and the eloquent location of her radiation field, it was decided that a low dose of 1800 cGy in 200 cGy fractions would be delivered to the face to a depth of 5 mm. An electron energy of 6 MeV was chosen with a desired bolus thickness of 1 cm in order to deliver the prescribed dose both at depth and to the skin surface. Immobilization was also an important component of treatment setup to minimize the margins required in the design of the planning target volume. To adequately cover the involved sites, the bolus would necessarily be placed over nearly the entire face, under the mask, which would be intolerable for the patient due to difficulty with breathing and claustrophobia. Therefore, an alternative technique was explored to create an immobilization device that would effectively function as a uniform bolus that would be tolerable to the patient on a daily basis.

The patient presented for simulation for radiation planning and to acquire mask measurements. A computed tomography (CT) scan of the patient's head and neck was obtained from crown to the hyoid bone for the fabrication of a custom face mask. The CT scan was reconstructed into 0.625 mm slices to provide a high resolution image. The Orfit (Orfit Industries, Antwerp, Belgium) head and neck immobilization device baseboard was measured with calipers to the nearest 0.01 mm and a virtual model was made using engineering software (SolidWorks 2008, Dassault Systèmes SolidWorks Corp., Concord, MA, USA). Lastly the neck pillow used was scanned using cone beam CT (ILUMA, IMTEC/3M) and a high resolution image was saved in.STL format.

**Figure 2:**
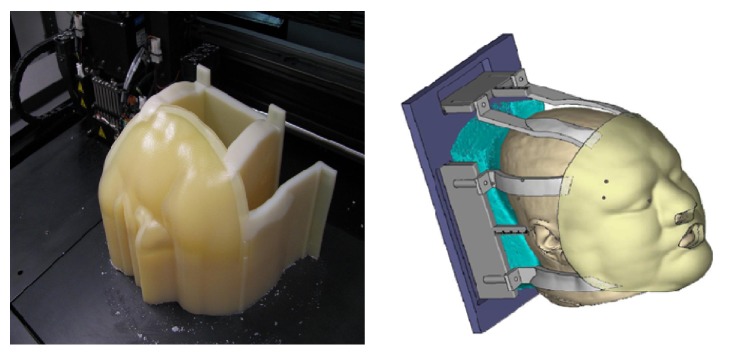
Fabrication of immobilization mold using customized bolus.

**Figure 3:**
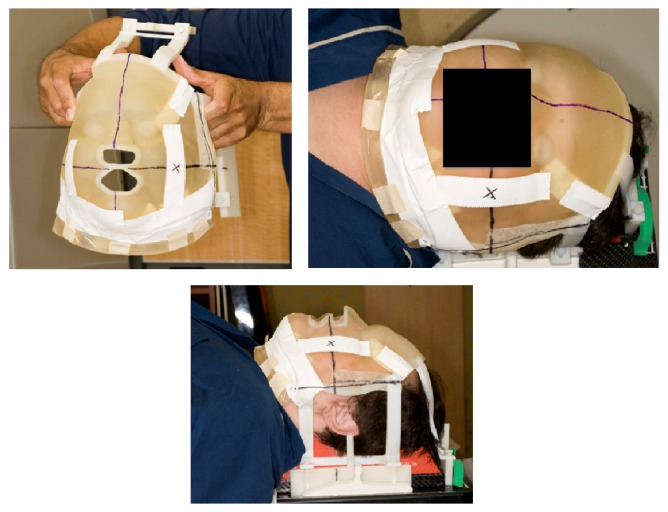
Final immobilization mold using customized bolus.

The separate virtual images of the patient, neck pillow, and immobilization device baseboard were combined into one model. The mask was designed using medical software (Mimics, Materialise, Ann Arbor, MI, USA) and coupled with the attachment apparatus designed on the virtual model (Figure 2). Using PolyJet printing technology (Connex500, Objet Geometries), the soft, pliable mask and rigid connecting apparatus were fabricated (Figure 3). The mask material was a proprietary “rubber-like” material (TangoPlus, Objet Geometries, Billerica, MA, USA) with a density of 1.141 gr/cm^3^. The patient returned for a second simulation appointment, at which time the mask and stabilization apparatus were fit to the patient.

The water equivalent thickness of the rubber material was studied using phantom measurements. A depth ionization distribution in polystyrene was measured for a 6 MeV electron beam using the Memorial Pipe chamber, a thin-window (0.05 mm) parallel-plate ionization chamber. Then, the measurement was repeated with a flat, 1.29 cm thick, sample of the material placed on top of the polystyrene. The offset between the two resulting depth ionization distributions represents the radiological equivalent thickness of the bolus material.

## 3. Results

**Figure 4:**
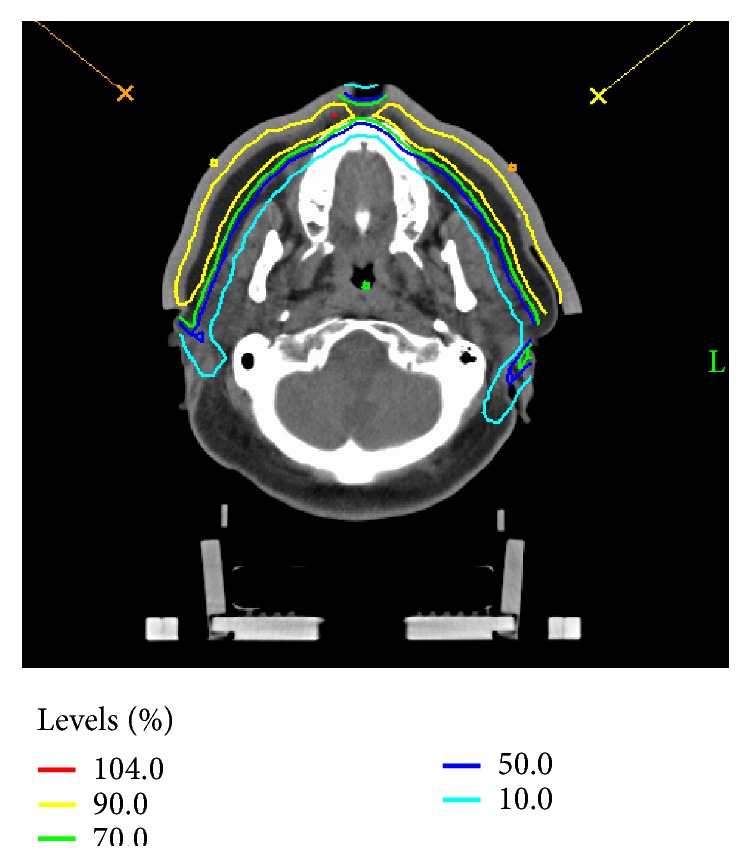
Dose distribution of 6 MeV electrons with customized bolus face mask in place; dose was prescribed to the 90% isodose line (yellow contour).

Figure 4 is a representative slice of the resulting treatment plan (Figure 4). A beam arrangement of two en face electron beams was chosen. One beam was directed towards each cheek using a source to surface distance of 105 cm. An in-house treatment planning system (TPS) was employed with dose calculations conducted via pencil beam convolution with a calculation grid size of 2 mm. A gap between the field edges was used at the skin surface along the midline of the face such that hot spots that were deemed intolerable were minimized and the best possible prescription dose coverage throughout the entire face was maintained.

From measurement, the radiological equivalent thickness of the bolus was equal to 1.024 times the geometric thickness. The measured HU in the TPS was 100, and calculations within the TPS result in a radiological equivalent bolus thickness of 1.08 cm. This indicates that the TPS will predict the penetration depth of the electron beam within approximately 0.5 mm.

**Figure 5:**
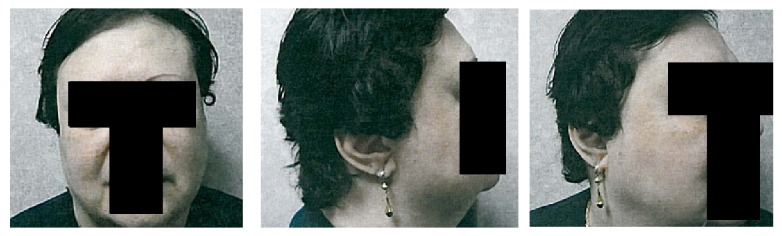
Follow-up of patient at 4 years demonstrating excellent cosmesis.

The intaglio surface of the mask was cleaned daily with mild soap and water as the material does not breathe well and the patient perspires during treatment. The mask maintained integrity throughout daily treatment with no evidence of stretch or deformation. The patient sustained mild grade I erythema during the course of radiation which resolved within several weeks after completion of her radiation course. Her facial lesions sustained a complete response at four-year follow-up with no evidence of radiation-related sequelae (Figure 5).

## 4. Discussion

Traditionally and currently in clinical practice, lupus can be a relative contraindication for patients to receive radiation therapy. Here we demonstrate that radiation therapy can be performed in patients who require it using innovative approaches and should not be avoided altogether. For the patient we presented in this report, she had no other treatment options. Rather than assuming that radiotherapy could not be done safely and effectively, we used an innovative approach to utilize radiotherapy, which was effective (as evidenced by no progression of disease) and safe (as evidenced by no manifestation of skin toxicity after RT).

In this report, we describe an approach to the development of an immobilization device with integrated bolus used to develop a plan in a TPS. Widely available current techniques utilize a normal thermoplastic mask for immobilization and a commonly used bolus material. In this patient, for example, there would have been concerns with the use of a thermoplastic mask with supplemental SuperFlab. Sheets of SuperFlab cannot stay tight in all of the crevices of the face such as the eyelid and nasal ala, leading to localized air gaps and subsequent hot and cold spots. Since we are treating the “whole face,” the concerns with dose homogeneity with this more commonly used technique are more exaggerated than the typical patient. The technique using a thermoplastic mask in addition to PlayDoh could also pose a problem as PlayDoh is very difficult to mold in a uniform thickness while avoiding internal air bubbles. Also, it would be difficult to avoid compressing and reshaping the mold with repeated applications and thus, reproducibility may be compromised. The technique we describe brings a level of precision unavailable in most treatment techniques. Use of a digital patient model also avoids the local deformations in the face that can occur when a thermoplastic mask is held tight while it dries or when creating a moulage.

An alternative plan would have been to produce a stone cast of the patient from a facial moulage and fabricate the mask in medical grade silicone by hand. The advantages to a moulage are cost and the shorter mask production turnaround time. However, the moulage has inherent inaccuracies due to the physical material properties of expansion or contraction and the actual weight of the impression that can distort soft tissues. After the moulage is made and the stone cast poured, the mask must be designed by hand which can lead to a nonuniform thickness of bolus leading to hot/cold spots in the treatment plan. Additionally, even if the thickness is precisely controlled, silicone manipulated by hand will contain air bubbles thus leading to a nonuniform density and again causing hot/cold spots. By utilizing CAD/CAM technology, the above factors are controlled as the imaging does not distort the soft tissues of the face. Additionally, the machines that manipulate the material and fabricate the shape are extremely accurate and control for factors such as expansion/contraction, density, and thickness.

The amount of time spent in the design, fabrication, and quality assurance of this device can be broken into two parts: device-specific and patient-specific parts. The device-specific aspects are time consuming in which detailed measurements of the immobilization device base plate and head rest need to be taken and modeled in the CAD software. Further, the water equivalent thickness of the bolus material has to be determined. However, this only has to be done during initial commissioning of the system or if there are any modifications to the materials. The patient-specific aspect requires 2 CT scans, a high resolution scan to get the model of the patient's face and a subsequent scan to verify how well the mask fits and to verify its thickness. The fabrication of the mask itself is either performed by a third-party service or, as 3D printers become more widely available, in-house.

## 5. Conclusions

The possibility that radiation may be withheld from patients for whom it may be curative is concerning. This report describes an innovative technique that may be used to minimize risks to a patient with a complicated clinical scenario and raises awareness to a clinical practice trend in which patients with CVD may receive suboptimal care despite limited data [12, 13]. In this report, we describe an approach to the development of an immobilization device with integrated bolus for a difficult treatment site with excellent clinical results.
